# DNA Damage and Apoptosis as In-Vitro Effect Biomarkers of Titanium Dioxide Nanoparticles (TiO_2_-NPs) and the Food Additive E171 Toxicity in Colon Cancer Cells: HCT-116 and Caco-2

**DOI:** 10.3390/ijerph20032002

**Published:** 2023-01-21

**Authors:** Margherita Ferrante, Alfina Grasso, Rossella Salemi, Massimo Libra, Barbara Tomasello, Maria Fiore, Chiara Copat

**Affiliations:** 1 Department of Medical, Surgical and Advanced Technologies “G.F. Ingrassia”, University of Catania, Via Santa Sofia 87, 95123 Catania, Italy; 2 Department of Biomedical and Biotechnological Sciences, University of Catania, Via Santa Sofia 83, 95123 Catania, Italy; 3 Section of Biochemistry, Department of Drug Science, University of Catania, 95125 Catania, Italy

**Keywords:** TiO_2_, E171, metallic nanoparticles, HCT-116, Caco-2, apoptosis, genotoxicity

## Abstract

This study investigated the DNA damage and apoptosis in colon cancer cells HCT-116 and Caco-2 induced by engineered titanium dioxide nanoparticles (TiO_2_-NPs) (60 nm) and titanium dioxide food additive E171. MTT assays showed that both chemical forms significantly reduced cancer cell viability in a dose-dependent manner. In particular the food additive E171 induced a pronounced inhibitory effect on the growth of HCT-116 and Caco-2 cell lines (E171 IC50: 3.45 mg/L for HTC-116 and 1.88 mg/L Caco-2; TiO_2_-NPs 60 nm IC50: 41.1 mg/L for HTC-116 and 14.3 mg/L for Caco-2). A low level of genotoxicity was observed in Caco-2 cells, especially when treated with TiO_2_ 60 nm. Western blot analysis showed that HCT116 and Caco-2 treated cells did not overexpress apoptotic markers such as cleaved Caspase 3 and cleaved Parp. Moreover, further analysis by quantitative real-time PCR (qRT-PCR) showed that TiO_2_-NPs and E171 did not promote the expression of Bax or downregulation of Bcl-2, nor did they increase the Bax/Bcl-2 ratio. The assay data provide clear evidence that TiO_2_ can cause DNA damage but does not induce apoptosis or decrease long-term cell proliferation. In addition, the results show that E171 has a slightly higher level of cytotoxicity and genotoxicity. This suggests that exposure to E171 may be hazardous to health and that further research on biological effects is needed to promote safer practices in the use of this compound.

## 1. Introduction

Nanoparticles (NPs) are important components of the biogeochemical system, but their natural cycle has been altered by the introduction of artificial nanoparticles [1]. In fact, NPs have achieved incredible success in more and more fields, such as pharmaceutical, cosmetic, biomedical, food, automotive, and military industries, due to their special properties such as resistance, reactivity, electrical conductivity, and incredible flexibility [2]. Food additives with a nanoparticle structure are an important component of processed foods. As a consequence, consumers have been expressing concern about their potential adverse health effects for some time [3] and want to be better informed about their potential health consequences.

Our study focuses on titanium dioxide (TiO_2_-NPs), one of the most common nanoparticles used as a white pigment and opacifier in paints, pharmaceuticals, cosmetics, and foods [4]. It occurs in three different varieties: as rutile, anatase, and, less commonly, as brookite [5]. TiO_2_ is also used as a food additive that has no nutritional value and is listed as E171 to provide a white color or to tint other pigments. It was approved by the European Union in anatase and rutile in uncoated form and is not allowed to exceed 1% of food weight, according to the Food and Drug Administration (FDA) [6]. It is estimated that about 4 million tonnes of E171 are consumed worldwide each year [7]. Specifically, as a food additive, E171 consists of approximately 40% nano-sized TiO_2_ particles (<100 nm) and 60% micro-sized TiO_2_ particles (>100 nm). It is not a nanomaterial, according to European Commission Recommendation 2011/696/EU, which defines a nanomaterial as a compound containing more than 50% nanoparticles. The uncertainties regarding the identity and characterization of E171 were highlighted, and it was noted that no particle size limits for E171 were set in the EU specifications. EFSA noted that more data are needed, information on the particle size distribution of E171 as well as information on the percentage (in number and mass) of nanoscale particles, which is only possible after accurate detection and characterization by an appropriate analytical method. Although it has long been considered safe due to its low solubility, toxicity, and inertness [8], the latest EFSA safety assessment concluded that E171 can no longer be considered safe for use as a food additive [9] due to several uncertainties regarding its genotoxic potential.

For all the above-mentioned reasons, the use of E171 in food has become a topic of discussion. In particular, special focus has been given to the proportion of nano-sized particles (<100 nm), as there is evidence that they can be distributed through the bloodstream or lymphatic system, alter the intestinal barrier, and accumulate in organs [10], with TiO_2_ ultrafine particles being better absorbed by organs than fine particles [11,12,13], although the fate of NPs in the body may differ according to the size and surface charge [14]. Over the last year, a growing number of studies have investigated the effects of E171 and identified several potential adverse effects [15]. Little is also known about TiO_2_-NPs metabolism in our bodies. However, it seems that these molecules manage to be absorbed in a dose-dependent manner from the gastrointestinal tract and enter other organs [16]. Studies in rats and mice have shown that nanoparticles can cross the intestinal barrier, accumulate in the gut and cause paraneoplastic lesions [17], promote anxiety, increase the number of adenomas in the colon, induce hypertrophy and hyperplasia of goblet cells [18], and disrupt the composition and function of the gut microbiota. Despite several toxicological studies conducted in recent years, a robust risk assessment of oral exposure to E171 has not yet been satisfactorily achieved.

Human exposure to TiO_2_-NPs may occur during both manufacture and use. Inhalation is thought to be the main route of TiO_2_-NPs exposure in the workplace, followed by dermal exposure. Dermal exposure is also important for customers as TiO_2_-NPs are the most common nanomaterial in skin care products [8]. The oral route is the least studied, although E171 is widely used. Furthermore, in addition to food-grade titanium, attention must be paid to titanium that unintentionally enters the environment. The anatase form of TiO_2_ is most commonly used in food and is the main source of exposure for the general population [19]. In general, children appear to be the most exposed group, as they have lower body weight and consume more products containing E171 [15]. Indeed, little is known about the potential bioaccumulation of TiO_2_-NPs along the food chain and the dietary intake dose to the general population.

Taking into account all the doubts expressed in the scientific literature, and in accordance with the recommendations of EFSA, the aim of this study was to investigate the toxicological effects of E171(food grade—anatase) against TiO_2_-NP (60 nm reference material—rutile) on colon cancer cells, Caco-2 and HCT-116, with a particular interest in genotoxic effects that may lead to carcinogenic effects.

## 2. Materials and Methods

### 2.1. Preparation and Characterization of Stock Suspensions

TiO_2_-NPs standard (60 nm TiO_2_ Nano Powder, rutile, 99.9%, AEM) was purchased from Nanovision (Brugherio, MB, Italy), while E171 (anatase) was obtained from an Italian supplier of food coloring for bakeries.

Particles were weighed into 50 mL polypropylene vials, suspended in cell culture medium at a concentration of 1000 mg/L, and sonicated at 300 W for 15 min to prevent aggregation immediately before the dilutions required for cell exposure. In this way, three independent replicates were prepared for TiO_2_-NPs standard and E171 to allow the characterization of nanoparticles in solution.

The characterization of the number of particles/mL, size, and size distribution was carried out using a spICP-MS (NexION^®^ 350D, Perkin Elmer, Waltham, MA, USA). The instrument parameters and the operational conditions are the same given in a previous paper [20].

Transport efficiency was calculated using a certified reference material PELCO (Ag-NPs, 39 ± 5 nm, 110 ng/L, monitoring *m*/*z* 107), obtaining values ranging from 2.7 to 5.54%.

In cell culture medium, the size distribution of TiO_2_-NPs 60 nm showed a size range of 44–85 nm with a mean size of 66.2 ± 3.0, a most frequent size of 56.6 ± 4.4, and a particle concentration/mL of 4.1 × 10^12^ ± 6.2 × 10^8^; while for E171 we obtained a size range of 40–360 nm with a mean size of 150.0 ± 20.5 and a most frequent size of 176.3 ± 24.4 and a particle concentration/mL of 0.12 × 10^12^ ± 3.5 × 10^8^. In E171, the percentage of nanoparticles <100 nm was 32.1%, and the percentage of microparticles was 67.9%.

All stock suspensions were freshly prepared before each experiment.

### 2.2. Cell Culture and Treatment

Human colon cancer Caco-2 and HCT116 cells were obtained from the American Type Culture Collection (ATCC) (Rockville, MD, USA). The cell lines were cultured in RPMI-1640 medium supplemented with 10% fetal bovine serum (FBS), 100 IU penicillin, 2 mmol/L L-glutamine, and 100 mg/mL streptomycin and incubated at 37 °C in a humidified incubator containing 5% CO_2_. Mediums and all the supplements were provided by Lonza (Walkersville, MD, USA).

A dispersing agent such as FBS is needed to stabilize and disperse E171 and TiO_2_-NPs, as already shown for different nanomaterials, including TiO_2_ [21], and has been applied in larger harmonized studies on nanomaterials genotoxicity.

Cells were seeded in 25 cm^2^ flasks with vent cap and then passaged by 1:10 ratios using trypsin–EDTA 0.05% every 72 h. For all the experiments, the cells were trypsinized and sub-cultured in a cell culture medium supplemented with TiO_2_ (E171, 60 nm) according to the experiment selection.

### 2.3. Cell Viability and Proliferation

The viability of two cancer cell lines was assessed by the MTT assay. The Caco-2 and HCT-116 were seeded in 96-well plates in 200 μL of complete RPMI-1640 medium at a density of 5.0 × 10^3^ and 3.0 × 10^3^ cells per well, respectively, and incubated for 24 h. After exposure for 72 h with increasing doses of E171 and TiO_2_ 60 nm (0.001; 0.01; 0.1; 1; 10; 100; 1000 mg/L), cells were incubated at 37 °C in 5% CO_2_. After removing the medium, 100 μL of MTT working solution (5 mg/mL in RPMI) was added to each well for 4 h at 37 °C in a humidified incubator. Then 100 μL of the solution of 2-propanol and hydrochloric acid (50 mL + 167 μL) was supplied to each well to solubilize formazan blue crystals. A cell-free system was used to exclude NPs interference with the test reagents, in which only the NPs were incubated with the test reagents, and their absorption was monitored.

The absorbance was measured at 590 nm with the ELISA Tecan Sunrise Reader according to the manufacturer’s instructions. Cell viability was calculated as follows:

Cell viability percentage = [(sample OD − blank OD/control OD − blank OD)] × 100

### 2.4. Western Blot

All cells were cultured at a density of 1 × 10^6^ cells/well in 6-well culture plates and incubated for 24 h at 37 °C and 5% CO_2_. Cells were seeded with various concentrations of E171 or TiO_2_ 60 nm (0.1; 1; 10; 100; 500 mg/L) for 72 h. Cells without TiO_2_-NPs and puromycin 0.5 µg/mL were used as positive controls for apoptosis. Cells were then trypsinized with trypsin EDTA 0.05% and centrifuged.

Proteins were extracted from cellular pellets lysed in cell lysis buffer (NP40) (150 mM NaCl, 1.0% NP40, pH 8.0 50 mM Tris), supplemented with protease and phosphatase inhibitors (Roche Diagnostics, Indianapolis, IN, USA). After the lysis, the supernatant enriched with interest proteins was centrifugated. The Quick Start TM Bradford 1X Dye Reagent (Biorad Laboratories, Inc., Hercules, CA, USA) assay was performed on well-diluted proteins. This colorimetric assay exploits a dye, able to bind to alkaline residues of the proteins leading to a bathochromic shift from 465 nm to 595 nm. A standard curve was realized using increasing doses of bovine serum albumin (BSA) (0.3125, 0.625, 1.25, 2.5, 5, 10, and 20 μg/μL) and a blank with NP40 diluted 1:10 in water.

The sample’s protein concentration was determined by placing it in separate wells (containing 5 μL of diluted protein + 250 μL of dye reagent) with different dilutions of the protein extract. After incubation at room temperature for 5 min, the absorbance was measured at 595 nm with the Tecan Sunrise ELISA reader, and the concentration was calculated.

For each sample, 30 μg of proteins were separated through vertical electrophoresis using 4–15% Mini Protean TGX Precast Gels (cat. n. 4561083—Bio-Rad Laboratories, Inc., Hercules, CA, USA).

Bio-Rad Trans-Blot Turbo was used to transfer the gel proteins into a nitrocellulose membrane (Bio-Rad Laboratories, Inc.). The transfer of protein was assessed with the Red Ponceau dye. Afterward, the membrane was left for one hour in 5% semi-skimmed milk after being rinsed with TBS-T (0.1% Tween 20, 20 mM Tris–HCl pH 7.6, 137 mM NaCl).

Two apoptosis markers, cleaved Caspase-3 and total/cleaved PARP proteins, were detected using the anti-Cleaved Caspase-3 (Asp175) (5A1E) Rabbit mAb (Cell Signaling, #9664, Danvers, MA, USA) and Anti-PARP (46D11) Rabbit mAb #9532 (Cell Signaling) after being incubated overnight. The anti-beta Tubulin rabbit Ab (diluted 1:1000—cat. n. 15568-Abcam, Cambridge, UK) and the Anti-Histone H3 rabbit Ab (ab1791—Abcam, Cambridge, UK) were used to detect Tubulin and Histone H3 housekeeping proteins. Then, the membrane was rinsed thrice for 10 min using TBS-T solution and incubated for one hour at room temperature with anti-mouse or anti-rabbit secondary antibodies conjugated with horseradish peroxidase (HRP) (diluted 1:10,000). Again, the membrane was rinsed thrice by using a TBS-T solution.

The Clarity Western ECL Substrate (cat. n. 1705060—Bio-Rad Laboratories, Inc., Hercules, CA, USA) was used to detect the chemiluminescence. Western blot images were acquired by Bio-Rad ChemiDoc Touch Imaging System and then analyzed with ImageJ software (National Institutes of Health, Bethesda, MD, USA). All Western Blot experiments were performed in triplicate.

### 2.5. Clonogenic Assay

After 72 h exposure to TiO_2_-NPs, the Caco-2 and HCT116 were seeded at 200 cells per well onto 24-well culture plates for 7 days in complete RPMI-1640 media. After being washed twice with ice-cold PBS, cells were fixed with ice-cold methanol for 20 min.

Then methanol was removed from plates that were then rinsed with water. 0.5% crystal violet solution was added before incubating the plates at room temperature for 5 min. Finally, the plates were rinsed again with water. Colony-forming assays were performed at least twice in quadrupled.

### 2.6. Comet Assay

Genotoxicity analysis was assessed by standard alkaline comet assay on Caco-2 and HCT-116 treated for 72 h with either TiO_2_ nanoparticles or E171.

According to Tomasello et al. (2017), the cell suspension was mixed with 0.7% low-melting point agarose at 37 °C [22]. Next, the cell suspension was spread onto microscope slides pre-coated with 1% normal-melting point agarose, and two mini-gels were made on each slide. After the gels were maintained at 4 °C for 10 min, the embedded cells were lysed in a fresh lysis buffer (2.5M NaCl, 0.1M EDTA, 1% Triton X-100, 1% N-laurosyl sarcosine, 10% DMSO, pH 10) for 1h in the dark at 4 °C. DNA unwinding was allowed for 20 min in a fresh electrophoresis buffer (300 mM NaOH, 1 mM, Na2EDTA, pH = 13.1) followed by electrophoresis for 20 min (1 V/cm) in the same re-circulating pH 13.1 buffer. All the slides were neutralized for 3 × 5 min in 0.4M Tris, dipped in 70% ethanol, and air-dried overnight. DNA was stained with SYBR-green diluted in TAE buffer (1:10,000). A maximum of one hundred nucleoids per sample (50 for each of the two replicate slides) were randomly acquired using the Leica epifluorescence microscope. The DNA damage was quantified by CASP-free software, and the results were expressed as the average percentage of fragmented DNA in the comet tail (%TDNA).

### 2.7. Quantitative Real-Time PCR (qRT-PCR)

After 72 h of treatment with increasing doses of TiO_2_-NPs and E171 (0.1; 1; 10; 100; 500 mg/L) in HCT116 and Caco-2 cell lines, we determined the mRNA expression of apoptotic regulators such as Bax and Bcl-2.

Total RNA extraction was performed by Invitrogen^TM^ PureLink^TM^ RNA Mini Kit from treated cells according to the manufacturer’s instructions. The ratio of absorbance at 260 nm and 280 nm was studied using a Nanodrop 1000 spectrophotometer (Thermo Fisher Scientific, Mississauga, ON, Canada) to assess the RNA concentration and purity spectrophotometrically in molecular-grade water. Then, for each sample, 2 μg of total RNA were treated with DNase I, RNase-free (Cat. N. EN0525—Thermo Fisher Scientific TM) to remove possible DNA contamination. The cDNA was converted from the isolated total RNA by SuperScriptTM IV Reverse Transcriptase kit (Cat. N. 18090050—Thermo Fisher ScientificTM). In brief, 1 μg RNA from each sample was added to 50 μM Random hexamers, 2.5 mM dNTPs, and the reaction volume became 13 μL with RNase-free water and mixed gently. Next, the mixtures were incubated at 65 °C for 5 min. After, the mixtures were supplemented with 4 μL 5x Super Script Buffer, 100 mM DTT, 1 μL Super Script IV Enzyme, and RNase-free water to obtain 20 μL reaction volume. The whole was incubated at 23 °C for 10 min, 55 °C for 10 min to start the reverse transcriptase enzyme, and incubated at 85 °C for 5 s to block the reaction. After reverse transcription reactions, cDNA was applied for real-time quantitative RT-PCR on 7300 Real-Time PCR System (Applied Biosystems, Waltham, MA, USA). The RT-PCR was carried out in a final volume of 20 μL containing 10 μL SYBR green master mix, 50 ng cDNA, 0.18 μL forward primer (10 μM), 0.18 μL reverse primer (10 μM), and nuclease-free water to bring to volume (Luminaris Color HiGreen qPCR Master Mix, high ROX, Cat. N. K0362—Thermo Fisher Scientific TM).

Sequences of primers are provided in Table 1. The thermal cycling program for all target and reference genes was as follows: pre-denaturation (2 min. at 50 °C), denaturation (10 min. at 95 °C), annealing, and extension (15 s at 95 °C, 30 s at 60 °C, 30 s at 72 °C) for 40 cycles. The melting curve analysis condition was as follows: 15 s at 95 °C, 1 min at 60 °C, and 15 s at 95 °C. Duplicate experiments were carried out for each data set. GAPDH mRNA was amplified as a reference gene, and fold changes in each target mRNA expression were calculated relative to GAPDH mRNA expression via the 2−^ΔΔCT^ method.

### 2.8. Statistical Analysis of Data

GraphPad Prism 6.0 software was applied for statistical data analysis. The mean ± SD of three independent tests is shown for each group. *p* < 0.05 was considered meaningful. One-way analysis of variance (ANOVA) and Student *t*-test were performed to determine statistical differences among groups. Dunnett’s’ adjustment was used for multiple comparisons.

## 3. Results

### 3.1. Effects of TiO_2_ on HCT-116 and Caco-2 Cell Viability

The growth-inhibitory effect of two forms of TiO_2_ was examined in vitro against human colon cancer cells. All cell lines were treated with increasing concentrations of TiO_2_ nanoparticles. MTT assay was used to evaluate cell survival after 72 h incubation.

**Figure 1:**
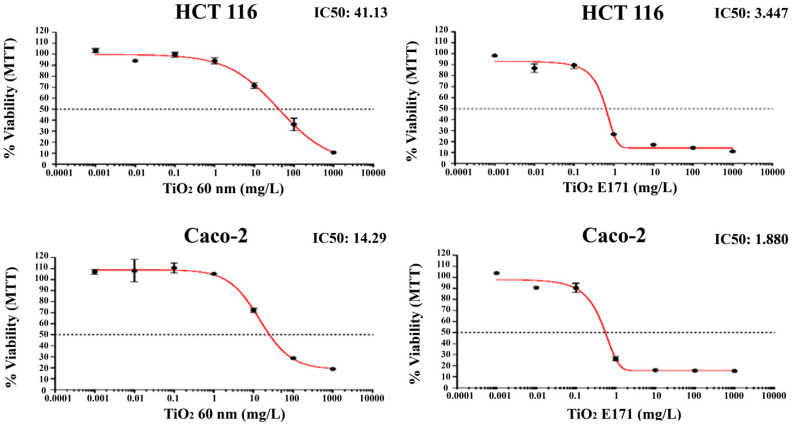
Inhibitory effect of TiO_2_-NPs and E171 on the growth of HCT-116 and Caco-2 cell lines. Cells’ viability evaluated through the MTT assay after 72 h of TiO_2_-NPs and E171 at different concentrations (0.0001; 0.001; 0.01; 0.1; 1; 10; 100; 1000 mg/L). Data are expressed as mean ± SD of three separate experiments.

The percentage of growth inhibition of increasing concentrations of TiO_2_-NPs and E171 against human HCT-116 and Caco-2 colon cancer cell lines is shown in Figure 1.

Both TiO_2_-NPs and E171 induce superimposable anti-proliferative effects in vitro and reduce the viability of HCT-116 and Caco-2 cell lines in a concentration-dependent manner.

Notably, the most pronounced anti-proliferative effect was induced by E171 on both cell lines. Indeed, the food additive E171 induced a peak collapse in the number of both cells with a reduction of more than 70% in the number of cells after exposure to 1 mg/L and of more than 80% with doses of 10, 100, and 1000 mg/L.

A lower cytotoxic effect was found after 72 h exposure to 60 nm TiO_2_-NPs. As shown in Figure 1, a significant reduction occurred in both cells, only starting from 10 mg/L. However, even with 60 nm NPs, the reduction of cell number appears to be dose-dependent, with only 10–20% of living cells after exposure to 1000 mg/L.

Finally, although both cells show a similar pattern in reducing cell viability, Caco-2 appears more resistant to higher concentrations.

It is notable that HCT-116 cells showed a lower decrease in cell viability for both treatments with TiO_2_ (TiO_2_ E171: IC50 = 3.447; TiO_2_ 60 nm: IC50 = 41.13) compared to Caco-2 cells (TiO_2_ E171: IC50 = 1.880; TiO_2_ 60 nm: IC50 = 14.29).

### 3.2. Measurement of DNA Damage by the Comet Assay

**Figure 2:**
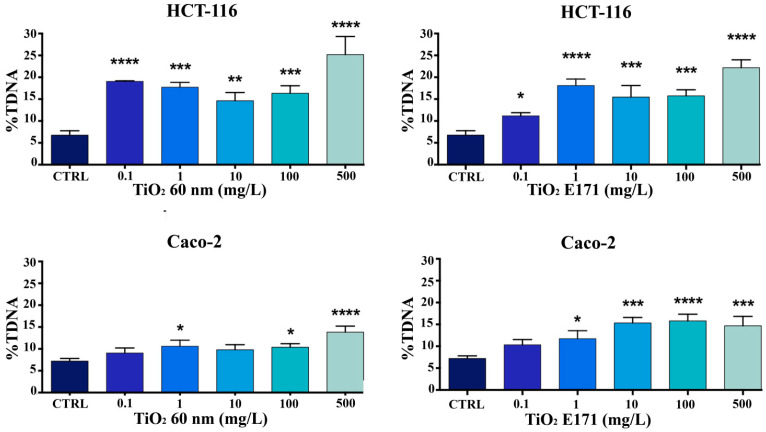
DNA damage was evaluated by alkaline comet assay. HCT-116 and Caco-2 cells were treated with different concentrations of TiO_2_ 60 nm and E171 for 72 h and investigated by alkaline comet assay. The results were expressed as the percentage of DNA present in the comet tail (%TDNA) and reported as mean ± SD. * *p* < 0.05 vs. control; ** *p* < 0.01 vs. control; *** *p* < 0.001; **** *p* < 0.0001 vs. control.

The genotoxic potential of TiO_2_ (E171 and TiO_2_ 60 nm) was evaluated using comet assays after 72 h of exposure. The %TDNA-the relative DNA content of the comet tail was used for quantification. A significant concentration-dependent increase in DNA damage was observed in HCT-116 and Caco-2 cells at the higher TiO_2_ concentrations (100 and 500 mg/L) after 72 h of exposure. Overall, a higher sensitivity of HCT-116 compared to Caco-2 cells can be observed. As shown in Figure 2, we observed significant DNA damage in HCT-116 cells exposed to the high concentrations of the two compounds.

A low level of genotoxicity was observed in Caco-2 cells, as shown in Figure 2, especially when treated with TiO_2_ 60 nm. This result seems to confirm what was observed in the MTT assay and underlines that Caco-2 seems to be more resistant.

### 3.3. Effect of TiO_2_ NPs on Apoptosis-Associated mRNA Expression

**Figure 3:**
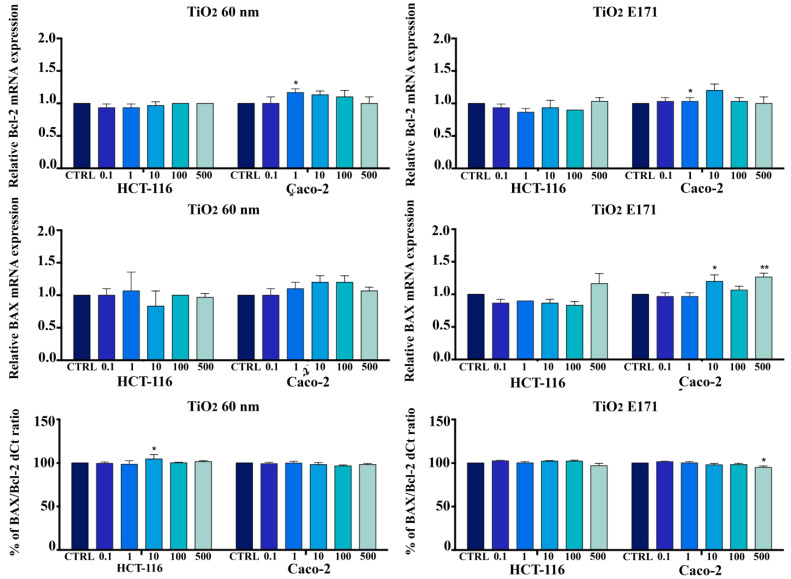
Bcl-2 and BAX mRNA expression: untreated vs. treated (72 h). Relative gene expression (mean fold change) of Bcl-2 and BAX in TiO_2_-treated versus non-treated HCT-116 and Caco-2 cells. Both cell lines were cultured with increasing doses of TiO_2_ (0; 0.1; 1; 10; 100; 500 mg/L) for 72 h, and then RT-PCR was carried out with specific primers. For analysis, GAPDH was used as the internal reference, and non-treated cells were used as the calibrator. mRNA relative expression for all genes was calculated by the comparative quantification Ct method (ΔΔCt). Results are representative of three independent experiments. * *p* < 0.05 vs. control; ** *p* < 0.01 vs. control.

The mRNA level of apoptotic markers (Bax, Bcl-2, and GAPDH) by RT-PCR was measured in cells (HCT116 and Caco-2) exposed to an increased concentration of TiO_2,_ as shown in Figure 3.

The comparative differential expression of selected genes was examined by SYBR-Green Real-time PCR in TiO_2_-treated HCT-116 and Caco-2 cells compared to non-treated cells. Expression levels of each gene were normalized with GAPDH Ct values.

After 72 h of treatment with increasing doses of TiO_2_-NPs and E171 (0.1–500 mg/L), in HCT116 and Caco-2 cell lines, the expression of Bcl-2 and BAX did not appear to be a critical marker of response to TiO_2_-NPs and E171 (Figure 3).

While TiO_2_ were able to decrease cell viability in both HCT-116 and Caco-2, they did not promote the upregulation of Bax or the downregulation of Bcl-2. They did not lead to an increase in the Bax/Bcl-2 ratio, as expected.

### 3.4. TiO_2_ Do Not Promote Apoptotic Cell Death in Cancer Cells

To further investigate the anti-proliferative effects of TiO_2_, we tested whether the two TiO_2_ forms could promote apoptotic cell death. We performed a Western blot experiment to assess the expression of two different apoptotic markers, cleaved caspase-3 and total/cleaved PARP proteins. A treatment with Puromycin was added to determine a positive control (CTRL) of cell death for apoptosis to ascertain the proper functioning of the antibody used.

**Figure 4:**
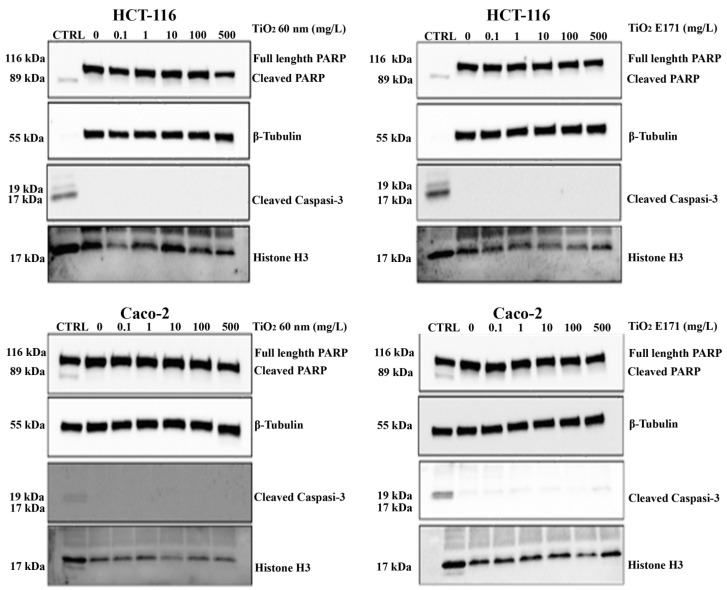
Representative images of apoptotic markers in colon cancer cell lines (HCT-116 and Caco-2) after 72 h exposure to E171 or TiO_2_ 60 nm.

As shown in Figure 4, we did not observe overexpression of either marker after 72 h of exposure to TiO_2_-NPs and E171. At all concentrations used, the expression of total PARP was higher than the control, while the expression of cleaved PARP and cleaved caspase-3 downstream of the apoptotic pathway was lower than the control.

Thus, the analysis of the protein expression of two markers of apoptosis (cleaved Caspase-3 and total/cleaved PARP) confirmed that the treatment with TiO_2_ did not induce apoptosis.

### 3.5. Clonogenic Assay

To investigate whether TiO_2_-NPs and E171 can slow down the proliferation of cancer cells after treatment, a clonogenic assay was performed.

**Figure 5:**
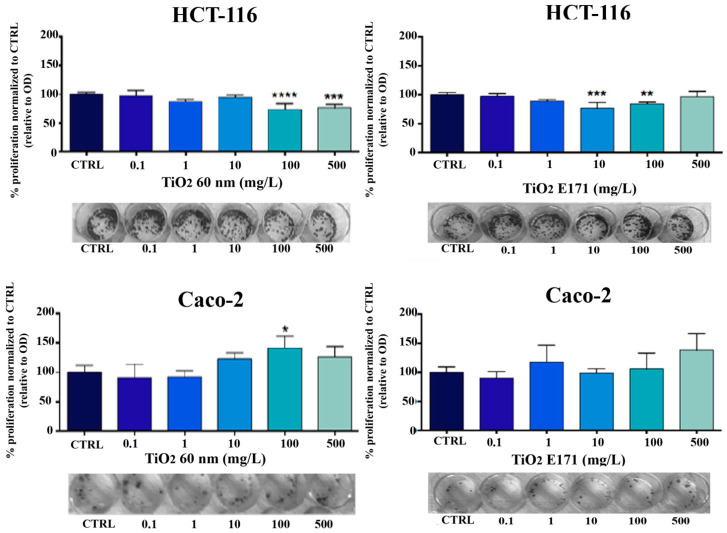
Clonogenic assay: HCT-116 and Caco-2 cell lines, 72 h post-treatment with TiO_2_-NPs and E171 (0; 0.1; 1; 10; 100; 500 mg/L), were seeded at 200 cells per well onto 24-well culture plates and allowed to grow for one week in complete RPMI-1640 media. The cells treated with TiO_2_ were compared to untreated cells (CTRL). * *p* < 0.05 vs. control; ** *p* < 0.01 vs. control; *** *p* < 0.001; **** *p* < 0.0001 vs. control.

The results showed that both cells proliferated again after treatment with TiO_2_-NPs and E171 and that cell growth was comparable to that of the control (Figure 5). Although a minimal reduction in cell growth was observed in the HCT-116 cell line at higher doses, neither form of TiO_2_ could slow the growth of the cancer cells, which is normally rapid.

## 4. Discussion

Several authors have raised concerns about the possible risks of oral intake of food-grade TiO_2_ E171 and TiO_2_-NPs. However, the underlying mechanisms of these adverse effects are not yet fully understood [23,24]. Nano-sized TiO_2_ is more toxic than larger particles [25], as small NPs have a larger total surface area, a higher total particle number, different shape, aspect ratio, and charge, which may lead to increased toxicity [26]. However, the biokinetic behaviors of TiO_2_ were found to be independent of particle size but highly affected by the presence of biomatrices [7].

In this study, we investigated the in vitro genotoxicity and apoptosis of TiO_2_-NPs 60 nm (rutile) and TiO_2_ E171 (anatase) in colon cancer cells: HCT-116 and Caco-2. Both are colon cancer cell lines that have been used in many in vitro studies since the 1980s. Although these cells originate from colon carcinoma, they differentiate when cultured under certain conditions and simulate the normal enterocytes of our intestine [27]. The current results show that TiO_2_-NPs and E171 had a cytotoxic effect on Caco-2 and HCT 116 cells in a concentration-dependent manner, indicating a possibly harmful effect, with a statistically significant effect from 1 mg/L for E171 and from 10 mg/L for TiO_2_-NPs 60 nm. This is in accordance with the results of the work done by other researchers [13].

Some studies reported that differentiated Caco-2 cells were found to be more resistant than undifferentiated Caco-2 cells [24], while E171 demonstrated toxicity in Caco-2 cells but not in HCT116 cells after 24 h exposure to 1000 μg/mL [28].

Both cells were found to be more resistant to 60 nm TiO_2_-NPs than to E171, as evidenced by the fact that the IC50 was approximately tenfold higher after exposure to 60 nm TiO_2_-NPs. This could be because the two forms of TiO_2_ studied have different crystalline structures, which may lead to different toxicity. In particular, the anatase form of TiO_2_ (E171) showed a slightly higher effect on cell viability than the rutile form of TiO_2_ 60 nm.

As reported in the literature, anatase TiO_2_ particles are significantly more potent than rutile in producing adverse biological effects, such as cytotoxicity, inflammatory responses, and the formation of ROS in a variety of cell types and tissues [29]. This is likely due to anatase being more chemically reactive than rutile [30], and it is well-known that the anatase phase benefits from nanosize due to its lower surface energy [31]. Furthermore, Proquin et al. (2017) have shown that a mixture of nano- and micro-sized TiO_2_ particles, such as those found in E171, cause more adverse effects than the individual fractions alone. This confirms the importance of testing food-grade TiO_2_ particles as a whole and not just their nano- and micro-fractions.

However, after removing the two forms of titanium, we found that cell proliferation returned to normal. For this reason, we hypothesize that the TiO_2_-NPs and food-grade TiO_2_ have a reversible cytotoxic effect by affecting the mitochondrial activity and metabolic function of cells, reducing cell viability but without deadly consequence. Several studies reported mitochondrial dysfunction characterized by the increase of mitochondrial reactive oxygen species (ROS), reduction in ATP generation, decrease in Krebs cycle metabolic flux, and cardiolipins synthesis [32,33,34].

It is also plausible that the deposition of the TiO_2_ on the well bottom inhibited cell growth due to a lack of space and/or limited cell adhesion to the well bottom. Our hypotheses were supported by the fact that the two pro-apoptotic cleaved proteins, caspase-3 and PARP, were unaffected by two TiO_2_ NPs, indicating a lack of apoptosis activation through this molecular pathway. This suggests that TiO_2_-NPs and E171 do not affect or are likely to reduce apoptosis. Similar results were reported by Kukia et al. (2018) [35], showing a reduction in apoptotic cells in a sample of HCT116 cells and a reduction in the Bax/BCL-2 ratio.

Two types of genotoxicity, primary and secondary, can be associated with TiO_2_ exposure. The first can be achieved through both direct interaction with DNA and indirectly by modulating the activity of proteins involved in cell processes (e.g., DNA repair or replication). The second form results from the overproduction of ROS arising due to various TiO_2_ mediated-mechanisms, including mitochondria dysfunction and inflammation [36]. The oxidative damage results in mutagenic events and accelerates pathophysiological conditions, including aging, inflammation, cancer, neurodegenerative diseases, and obesity [37].

The results of our comet assay showed that E171 and TiO_2_-NPs are genotoxic by inducing moderate DNA damage. Likewise, Proquin et al. (2017) found in their work that TiO_2_ particles, such as those contained in the food additive E171, can induce the formation of ROS and DNA damage in the colon-derived cell lines Caco-2 and HCT116. The dose-independent DNA fragmentation we observed can be explained by the direct binding of TiO_2_-NPs with the phosphate residues of DNA or can tuck themselves in DNA base pairs [38,39]. As a result of this direct interaction, DNA migration may be inhibited or slowed down. The genotoxic effect findings are also consistent with the safety assessment report for TiO_2_ (E171) published by the EFSA Panel on 6 May 2021. In this report, the Panel concluded that, considering the available evidence, a suspicion of genotoxicity cannot be excluded, and TiO_2_ can, therefore, no longer be considered a safe food additive. Furthermore, genotoxicity plays an important role in triggering carcinogenesis, as it can lead to alterations in genetic material, which in turn leads to changes in cellular signaling pathways related to cell proliferation and apoptosis [40]. However, it should be noted that the concentrations exhibiting cytotoxicity and genotoxicity are too high versus those derived from oral absorption of TiO_2_ from commercial products. Nevertheless, we should remember that the potential total amount of TiO_2_-NPs entering human tissues after oral ingestion might be influenced by the extreme conditions in our digestive system, which certainly affect particle size distribution, surface properties, and fate [41].

## 5. Conclusions

Overall, the results of the present study indicate that despite the impairment of cell viability by E171 and TiO_2_ 60 nm, both chemical forms of TiO_2_ do not induce apoptosis but have a genotoxic effect.

Furthermore, the resumption of cell proliferation after particle removal supports the notion that a better understanding of the biological effects of TiO_2_-NPs is needed to promote safer practices in the use of nanomaterials.

Further in vitro and in vivo studies need to be conducted to draw conclusions about the mechanisms behind the potentially carcinogenic effects of E171.

Furthermore, it is important to carefully study and analyze the physicochemical properties of TiO_2_ particles in their carrier as well as in the surrounding matrix as the final environment to ensure a sound evaluation of the potential adverse health effects of E171 and to adequately compare different studies in the context of risk assessment.

These findings could be relevant for the risk assessment of the food additive E171 and contribute to implementing the European Food Safety Authority (EFSA) risk assessment for the application of nanoscience and nanotechnologies in the food and feed chain.

## Figures and Tables

**Table 1:** Sequence of the primers applied for qRT-PCR.

Primer	Forward	Reverse
*Bax*	*5′-CCCCGAGAGGTCTTTTTCCG-3′*	*5′-TGGTTCTGATCAGTTCCGGC-3′*
*BCL-2*	*5′-TGAACTGGGGGAGGATTGTG-3′*	*5′-CGTACAGTTCCACAAAGGCA-3′*
*GAPDH*	*5′-AGAAGGCTGGGGCTCCATTTG-3′*	*5′-AGGGGCCATCCACAGTCTTC-3′*

## Data Availability

The data presented in this study are available on request from the corresponding author.
